# QuEChERS and HPLC-MS/MS Combination for the Determination of Chloramphenicol in Twenty Two Different Matrices

**DOI:** 10.3390/molecules24030384

**Published:** 2019-01-22

**Authors:** Tomasz Śniegocki, Bartosz Sell, Marta Giergiel, Andrzej Posyniak

**Affiliations:** Department of Pharmacology and Toxicology, National Veterinary Research Institute, 24-100 Pulawy, Poland; bartosz.sell@piwet.pulawy.pl (B.S.); marta.giergiel@piwet.pulawy.pl (M.G.); aposyn@piwet.pulawy.pl (A.P.)

**Keywords:** chloramphenicol, multi matrices, confirmatory method, QuEChERS, HPLC–MS/MS

## Abstract

A simple method for the determination of chloramphenicol in 22 matrices was prepared based on the QuEChERS and HPLC-MS/MS combination. Following a hydrolysis step, the homogenized samples were extracted and partitioned after adding sodium chloride with acetonitrile. Chloramphenicol was analysed by HPLC-MS/MS in negative electrospray mode by monitoring the daughter ions *m*/*z*: 321→194 and 321→152. The limit of decision (CCα) was calculated at the range of 0.10 μg kg^−1^ to 0.15 μg kg^−1^ and detection capability (CCβ) from 0.12 μg kg^−1^ to 0.18 μg kg^−1^. Validation results showed that this method is suitable for the determination and confirmation of chloramphenicol in various matrices.

## 1. Introduction

Chloramphenicol (CAP) is an inexpensive, antibiotic with broad-spectrum activity. The toxic effects of CAP for humans have resulted in restrictions on use in veterinary practice. A common side effect in humans is dose-dependent bone marrow suppression [1]. Due to the ban on the use of this substance in the European Union, a limit of 0.3 μg kg^−1^ as a minimum required performance limit (MRPL) was set [2,3]. CAP is rapidly metabolized and is eliminated via urine as a CAP glucuronide (CAPG)—the main metabolite [4]. That’s why simple, sensitive and fast methods for determination of CAP and CAPG residues are required. According to the literature, residues of CAP could be present also in processed foods such as cooked food [5], therefore it can be assumed that, the food which has been heated may also contain residues of CAP. Besides that, according to our experience, CAP could transfer from milk to milk products such as curd cheese, sour cream, butter and whey [6]. Also according to the results of research monitoring, CAP can occur in products such as meat, royal jelly, eggs, milk and milk products [7]. It is quite difficult to develop a simple method for such number of matrices because of a large variety of tested materials. These matrices are characterized by a very large variation due to the content of fat (butter, cream, eggs, feed), protein (sour cream, curd cheese, eggs, feed), water (water, whey, urine, muscle) and sugars (honey). Products may also contain added vitamins, oils, emulsifiers, flavor enhancers, salt and other spices. In addition honey is a difficult matrix because of its sugar composition which represents one of the most complex mixtures of natural carbohydrates and possible interfering substances. All of these different matrices may contain impurities which could have some influence on analysis with the use of HPLC and may lead to multiple overlapping peaks and a drifting background in mass spectrometry chromatograms [8].

Many analytical methods have been developed to determine CAP in multiple matrices, mainly using high performance liquid chromatography coupled with mass spectrometry (HPLC–MS) [1,6,8,9,10,11,12,13], but there are only a few papers about the determination of CAP in multiple matrices [1,6,8,9,10,11]. The purpose of this study was to create a simple, low-cost and fast method with good recovery and good purification of extracts by QuEChERS approach instead of LLE or classical SPE methods for confirmation and quantitation of CAP and CAPG (converted to CAP using the hydrolysis step) residues in 22 various matrices by HPLC–MS/MS system and validated according to Commission Decision 2002/657/EC criteria [13]. 

## 2. Results and Discussion

### 2.1. HPLC-MS/MS Conditions

**Figure 1:**
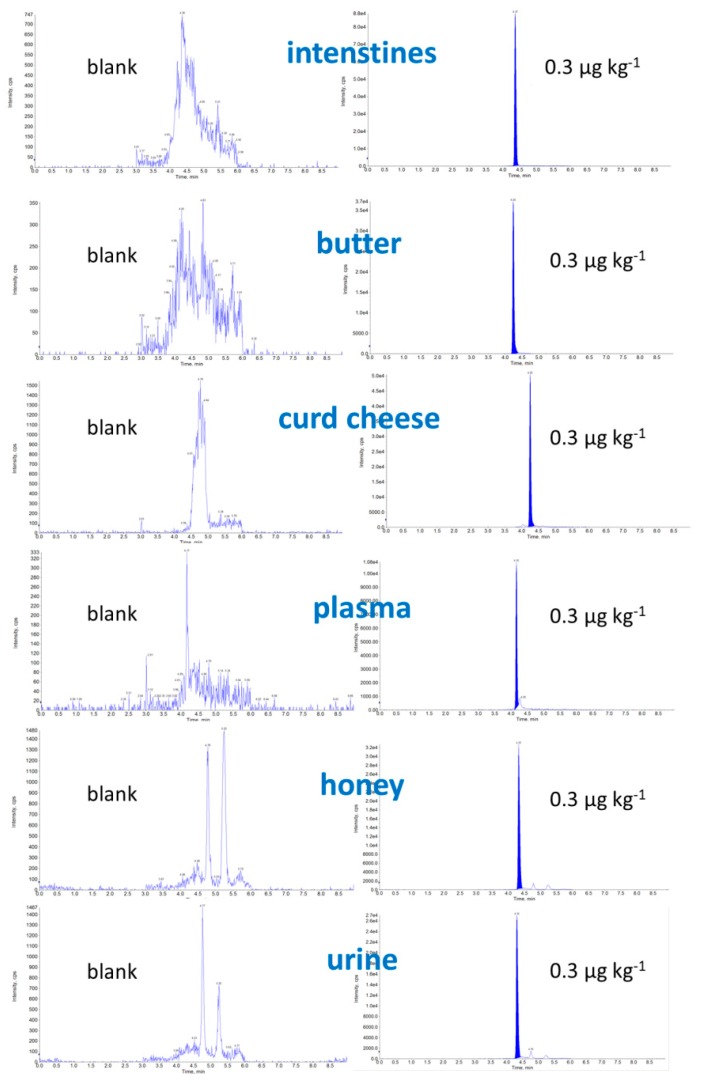
Example of chromatograms, blank and CAP spiked sample (0.3 μg kg^−1^).

Due to the large quantity of difficult matrices, we decided to not use our previously described mobile phase for determination of CAP, where CAP had a long retention time [14], but rather we applied previously described parameters from our method [8] including mobile phase and gradient modification with HPLC adaptation with a similar analysis time. The best separation (symmetric peak shape and minimal matrix effect) and longer retention time of the CAP were estimated with the use of 0.5% isopropanol in 0.1% acetic acid in water: methanol. Examples of chromatograms of a blank and fortified samples of intestines, butter, curd cheese, plasma, honey, and urine samples at the level of 0.3 μg kg^−1^ are shown in Figure 1. 

### 2.2. Optimization of Sample Preparation

The determination of chloramphenicol may be divided into the determination with the hydrolysis step (CAP is mainly in a glucuronic form (liver, kidney, urine, plasma)) and the direct determination of the parent drug (sour cream, milk, milk powder, butter, curd cheese, water, whey, eggs, eggs powder, aquaculture products, royal jelly, mead, honey, sausage, ham, headcheese, intestines, fat and feed). Based on experiments by Kittler et al. [15], we decided that the muscles matrix should be added to matrices where the hydrolysis step is needed. In the case of CAP in glucoronated form, the most frequently used enzymatic hydrolysis procedure involves the use of β-glucuronidase type HP-2 at pH 5.2, at 37 °C for 16 h, [1,16]. Due to the fact that this stage lasts 16 h, we decided that it should be optimized. To optimize the conditions, two parameters were used: temperature (37 °C, 45 °C, 50 °C, 55 °C, and 60 °C) and time (0.25 h, 0.5 h, 1 h, 2 h, 3 h, 4 h, 8 h, and 16 h).

**Figure 2:**
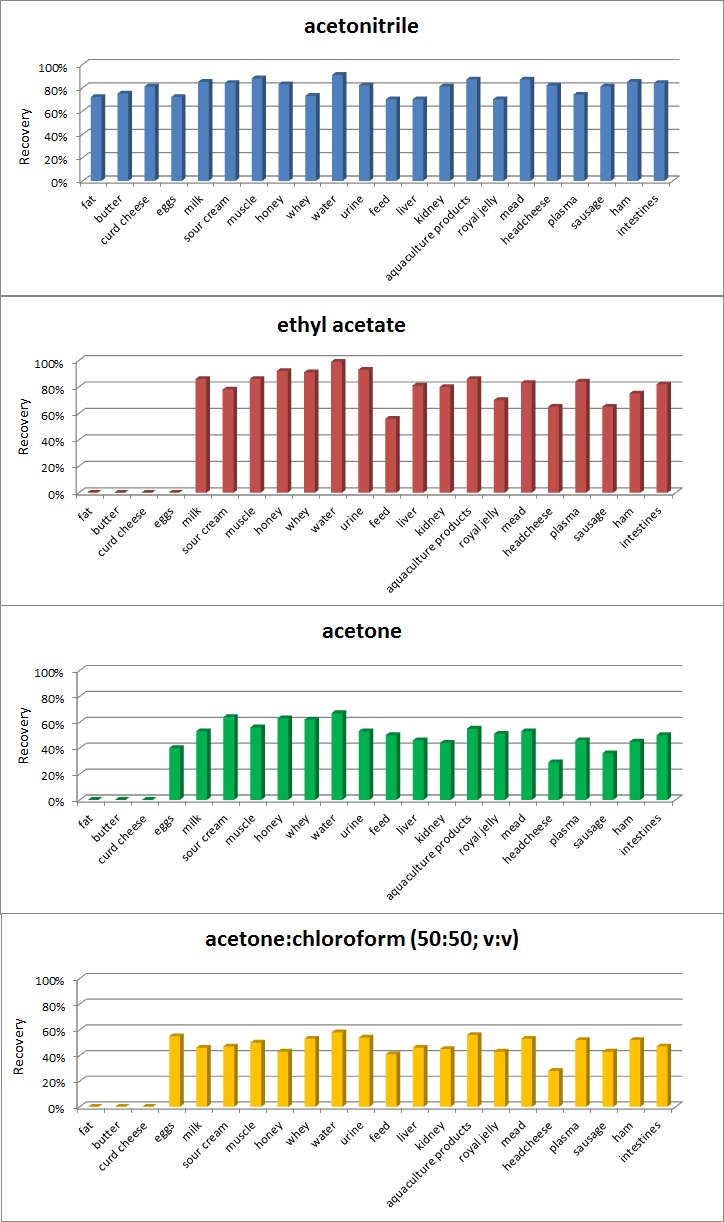
Comparison of suitability of different extraction solvents for chloramphenicol.

**Figure 3:**
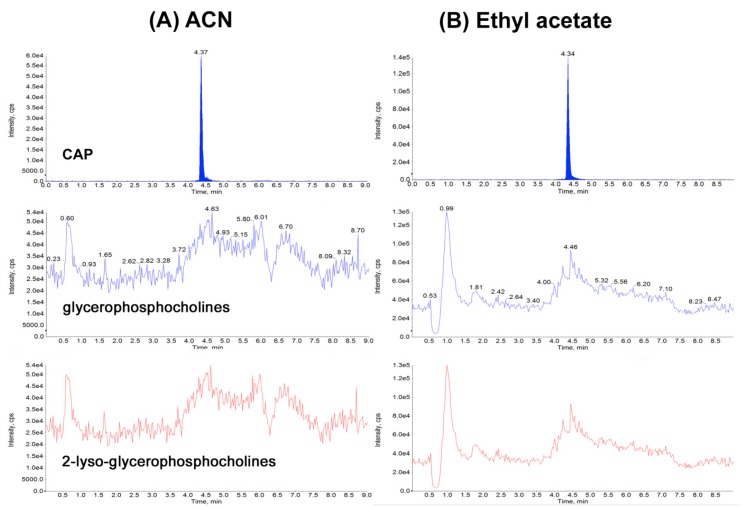
Chromatograms of chloramphenicol (1), glycerophosphocholines (184 *m*/*z*) (2) and 2-lyso-glycerophosphocholines (104 *m*/*z*) (3), extraction from muscle: (**A**) after acetonitrile extraction (**B**) after ethyl acetate extraction.

The most reproducible results, with the shortest time and the lowest temperature and when CAPG not detected were already obtained at a temperature of at least 50 °C and after 1 h. Many procedures describe the determination of CAP residues in biological matrices [1,6,8,9,10,11,12,13], but only a few are available for different kinds of food of animal origin matrices [1,6,9,10,11,12]. The purpose of this study was to create a simple, low-cost and fast method with good recovery and good purification of extracts by QuEChERS which is between LLE (simple, low-cost and fast) and SPE (good recovery and good purification of extracts) for confirmation and quantitation of CAP and CAPG residues in various matrices. Based on our study we decided to use acetonitrile for CAP analysis, because it is a compromise between recovery (Figure 2) and purity of extracts (the purity of the extract was measured by the amount of phospholipids interferences on the chromatogram after extraction step) for all tested matrices and solvents (Figure 3).

To compare the recovery, the samples were treated with ethyl acetate, acetonitrile, acetone, and chloroform:acetone (50:50), and then centrifuged. The worst results (recovery) were with a mixture of acetone:chloroform, especially in fat, feed, butter, and cheese. The large amounts of co-extractive matrix compounds prevented evaporation to dryness. Poor results were also obtained for eggs with ethyl acetate (emulsion). Acetone and mixtures of acetone:chloroform gave an equally poor recovery (Figure 2).

Determination of phospholipids was conducted based on the experiments by Flieger et al. [17]. We realize that for some matrices (muscle, aquaculture products, water, whey, liver, kidney, ham, headcheese, sausage, intestines, mead, royal jelly, honey, plasma and urine) better solution (recovery) could be ethyl acetate, but due to the maximum simplification of the procedure and purity of extracts (smaller amount of phospholipids Figure 3), we decided to use acetonitrile for extraction from all matrices. Other parameters for reagents (NaCl, C18 sorbent, PSA, anhydrous MgSO_4_) were selected based on our experience in selecting conditions for CAP determination in dairy products [6].

### 2.3. Validation Result

The whole procedure was validated according to 2002/657/EC decision on the quality standard [3]. The analysis of 20 blank samples for all matrices did not reveal any interference. The criteria concerning relative retention time of the analytes correspond to that of the calibration solution at a tolerance of ±2.5%. The CCα and CCβ values were also determined and presented in Table 1, which contains the summary of the data obtained from the validation process. The apparent recoveries were in the range of 93.1% to 108.0% with repeatability less than 9.2%, and within-laboratory reproducibility below 13.1% (Table 2).

The expanded uncertainty was calculated at the MRPL level [18] (Table 1). The calculation of ion suppression of the matrix effects for CAP for all matrices indicates that it is not a problem of this method (Table 1). Values of correlation coefficients obtained from plotting the peak area corrected by internal standard in relation to the nominal concentration were higher than 0.98 at each matrix. The parameters of the linear regression function are presented in Table 1. To check the occurrence of statistically significant differences for the sensitivity of the method for the tested matrices, the confidence interval was calculated. The t-student distribution for 28 degrees of freedom was used to calculate the confidence interval. Most of the matrices showed no statistically significant differences. However, statistically significant differences were found between whey and plasma, ham, intestines, aquaculture products and headcheese.

## 3. Experimental Section

### 3.1. Materials and Methods

Acetonitrile, isopropanol and ethyl acetate were obtained from Merck (Darmstadt, Germany). Primary and secondary amine (PSA) was purchased from Supelco (Belleforte, PA, USA). Sodium chloride (NaCl) and methanol were purchased from P.O.Ch. (Gliwice, Poland). CAP, CAP-D5 Anhydrous sodium sulphate (Na_2_SO_4_), anhydrous magnesium sulphate (MgSO_4_), and β-glucuronidase type HP-2 were from Sigma-Aldrich (St. Louis, MO, USA). Pre-heated magnesium sulphate (MgSO_4_) was prepared in our laboratory for heating (400 °C) overnight. Octadecylsilane sorbent (C18), ammonium acetate and acetic acid were obtained from J.T. Baker (Deventer, The Netherlands). All reagents were HPLC grade. Nanosep MF filter was supplied by Pall (Port Washington, NY, USA). Ultrapure water was filtered through a Millipore Milli-Q system (Burlington, MA, USA). Kinetex C8 column (75 mm × 2.1 mm × 2.6 μm) and C8 precolumn (4 mm × 2 mm × 4 μm) were obtained from Phenomenex (Torrance, CA, USA). Stock standard solution of CAP and CAP-D5 (1 mg mL^−1^) were prepared in methanol and stored in the dark into a 10 mL amber volumetric flask. This standard will expire in 12 months when stored at <−18 °C. The working standard and internal standard solutions at the level of 0.01 μg mL^−1^ were prepared in methanol and stored in the dark into a 5 mL amber volumetric flask. This standard will expire in 12 months when stored at <6 °C. 

For the procedure optimization and validation, milk, curd cheese, whey, butter, and sour cream with various protein content (1% protein in butter −60% protein in eggs) and various fat content (0.5% fat in whey −82% fat in butter), eggs, muscle (chicken, pig), liver, kidney, honey, sausage, ham, headcheese, intestines, aquaculture products (shrimp, fish) royal jelly and mead were collected from the supermarkets. The animal feed (pig, poultry) were collected from a feed producer. Water, plasma and urine were obtained from livestock. Samples have been checked so that they did not contain CAP residues. Thereafter, samples were stored at <−18 °C until the experiment.

### 3.2. HPLC-MS/MS

The HPLC-MS/MS system was composed of an ABSciex ExionLC HPLC system (Concord, ON, Canada) connected to ABSciex API 5500 Qtrap mass spectrometer. The Analyst 1.6.3 software controlled the HPLC-MS/MS system and Multiquant 3.2 to process the data. The MS system was operated in the electrospray negative ionization mode with a capillary voltage of 4.5 kV. The multiplier was set at 1900 V. The desolvation temperature was set at 500 °C, collision gas (N_2_)—high; nebuliser gas (N_2_)—36 psi; gas 1 (air)—35 psi; gas 2 (air)—35 psi; curtain gas (N_2_)—36 psi. The chromatography was performed on a Kinetex C8 column (75 mm × 2.1 mm × 2.6 μm), connected to a C8 precolumn (4 mm × 2 mm × 4 μm). The mobile phase for LC analysis was composed of two reagents: A (0.5% isopropanol in 0.1% acetic acid in water) and B (methanol). Composition of mobile phase (A:B, *v*:*v*) was started at 15% of B to 2.5 min, then 45% at 3.0 min, and held for 3 min, then 15% and held for 3 min. The equilibration time was 3 min. The column was operated at 40 °C at a flow rate of 0.4 mL min^−1^. The ions monitored by multiple reactions monitoring (MRM) were 321→194 and 321→152. The declustering potential (DP) was −105 eV. The optimised collision energy (CE) for CAP was −16 eV for first daughter ion and −22 eV for the second one. 

### 3.3. Sample Preparation

For urine, plasma and homogenised liver, muscle and kidney a 2 ± 0.05 g of sample were mixed with 30 µL of the working solution internal standard (CAP-D5), thereafter 1.5 mL of 0.05 M acetate buffer, (pH 5.2) and 50 µl of β-glucuronidase were added, and the sample was homogenised for approx. 60 s. The samples were hydrolysed by 1 h. in temp. 50 °C.

Intestine samples were first homogenised with liquid nitrogen and after that, 2 ± 0.05 g of the sample was mixed with 30 µL of the internal standard working solution (CAP-D5) and were mixed for 0.5 min on a vortex mixer at 349× rcf with 1.5 mL of water. 

For other matrices (sour cream, milk, milk powder, butter, curd cheese, water, whey, eggs, eggs powder, aquaculture products, royal jelly, mead, honey, sausage, ham, headcheese, fat and feed) a 2 ± 0.05 g homogenised sample with 30 µL of the internal standard working solution (CAP-D5) were mixed for 0.5 min on a vortex mixer at 349× rcf with 1.5 mL of water. 

After this step all samples were mixed with 10 mL of acetonitrile. Then, 0.5 g NaCl was added and mixed for 1 min on a vortex mixer at 349× rcf and centrifuged at 2930× rcf for 10 min at about 6 °C. The 7 mL of the top layer were transferred to new centrifuged tube, and subsequently 100 mg PSA, 200 mg C18 and 600 mg pre-heated anhydrous MgSO_4_ were added. Then extract was mixed for 2 min on a vortex at 349× rcf and centrifuged at 2930× rcf for 10 min at about 6 °C. The 5 mL of the top layer was transferred to new centrifuge tube and evaporated under gentle nitrogen stream at about 45 °C, residue was redissolved in 0.3 mL of 0.5% isopropanol in 0.1% acetic acid and centrifuged with Nanosep MF filters (0.22 µm) at 9447× rcf in room temperature for 10 min and transferred to autosampler vial for analysis.

### 3.4. Validation

The method was validated according to the requirements of the Commission Decision 2002/657/EC [3]. The validation parameters: selectivity, linearity, repeatability, within-laboratory reproducibility, recovery, uncertainty, decision limit (CCα), and detection capability (CCβ) of the method were estimated. In selectivity study, possible interferences encountered in the method have been checked by analysis of 20 blank samples for each matrix from different sources. The linearity was evaluated based on matrix-matched calibration curves, which were prepared by fortifying blank samples (for each matrix) at six con-centration levels corresponding to 0.1 (or 0.15 depending on the matrix); 0.3; 0.45; 0.6; 1.2; 2.4 μg kg^−1^ containing an internal standard (0.6 μg kg^−1^) [19]. The repeatability and reproducibility were determined at four concentration levels (six samples of each level) 0.1 (or 0.15 depending on the matrix); 0.3; 0.45; 0.6 μg kg^−1^. For matrices such as feed (pig, poultry), aquaculture (fish, shrimp) and muscle (chicken, pig), the samples in the validation were divided equally. For repeatability the samples were analysed by the same operators, on the same day with the same instrument and were calculated as the relative standard deviation (RSD, %). For within-laboratory reproducibility another two sets of blank samples were fortified and analysed by different operators, on another two days with the same instrument, and were calculated as the relative standard deviation (RSD, %). The recovery was calculated by comparing the mean measured concentration with the fortified concentration of the samples. Decision limits (CCα) and detection capabilities (CCβ) were calculated according to the approach described in the ISO standard and SANCO/2004/2726 [3,19,20]. Values CCα were calculated on the basis of decision 2002/657/EC and oscillated around 0.1 μg kg^−1^ and 0.15 μg kg^−1^ depending on the matrix. The SANCO/2004/2726 guidelines imposes an obligation to check these levels and establish the level of CCα as the lowest point on the curve that has been checked during validation [3,19,20]. Matrices were divided into the CCα value close to the level of 0.1 μg kg^−1^ and 0.15 μg kg^−1^. Then 20 samples were fortified for each matrix in order to determine the CCα level. The matrix effect was checked by analysing five different samples for each matrix at 0.3 μg kg^−1^ concentration level, which is the most important level for chloramphenicol and calculated by the equation proposed previously by Matuszewski et al. [21]. The expanded uncertainty was calculated at the 0.3 μg kg^−1^ concentration level by applying a coverage factor of 2, which gives the level of confidence of approximately 95% [18]. 

## 4. Conclusions

The validation study showed that, the presented method is a reliable confirmatory strategy for the determination of CAP and CAPG residue in all validated matrices. The CCα and CCβ values determined for CAP in twenty two matrices were below 0.3 μg kg^−1^ (MRPL). Application of the method was confirmed in proficiency tests like FAPAS (z-score = 0.3 for honey) and ANSES Fougeres (z-score = 0.37 for shrimp, z-score = −1.11 for urine, z-score = 1.47 and 0.7 for muscle) with satisfactory results with z-scores between −2.0 and 2.0. 

To the best of our knowledge, this is the first simple method based on the QuEChERS and HPLC-MS/MS combination reported for the total determination of CAP in various matrices based on the QuEChERS approach. The analytical method presented in this article could also be successfully applied to many other matrices not verified during validation. This method is used for the effective routine analysis and for official control of products in Poland.

## Figures and Tables

**Table 1:** Validation report.

Matrix	CCα [µg kg^−1^]	CCβ [µg kg^−1^]	Matrix Effect (%)	Expanded Uncertainty [µg kg^−1^]	Concentration Range (ng/mL)	Determination Coefficient	Calibration Curve
butter	0.10	0.12	8.2 ± 3.1%	0.30 ± 0.04	0.10–2.4	0.987	*y* = 0.8134 (±0.0412)*x* + 0.0123 (±0.0006)
sour cream	0.10	0.13	8.1 ± 2.7%	0.30 ± 0.03	0.10–2.4	0.992	*y* = 0.7425 (±0.0443)*x* + 0.0052 (±0.0003)
curd cheese	0.10	0.13	7.3 ± 3.0%	0.30 ± 0.04	0.10–2.4	0.982	*y* = 0.7525 (±0.0451)*x* + 0.0062 (±0.0004)
whey	0.10	0.12	3.2 ± 1.2%	0.30 ± 0.04	0.10–2.4	0.993	*y* = 0.7143 (±0.0283)*x* + 0.0062 (±0.0002)
milk	0.10	0.12	7.1 ± 2.2%	0.30 ± 0.04	0.10–2.4	0.995	*y* = 0.7498 (±0.0384)*x* + 0.0032 (±0.0001)
water	0.10	0.12	3.1 ± 1.2%	0.30 ± 0.03	0.10–2.4	0.998	*y* = 0.7604 (±0.0461)*x* + 0.0067 (±0.0004)
feed	0.15	0.18	11.1 ± 5.3%	0.30 ± 0.09	0.15–2.4	0.983	*y* = 0.7724 (±0.0312)*x* + 0.0228 (±0.0009)
urine	0.15	0.18	6.9 ± 2.1%	0.30 ± 0.06	0.15–2.4	0.985	*y* = 0.7424 (±0.0445)*x* + 0.0148 (±0.0009)
plasma	0.15	0.17	6.4 ± 2.5%	0.30 ± 0.04	0.15–2.4	0.982	*y* = 0.8750 (±0.0376)*x* + 0.0365 (±0.0016)
muscle	0.10	0.12	5.8 ± 2.9%	0.30 ± 0.05	0.10–2.4	0.997	*y* = 0.8654 (±0.0519)*x* + 0.0324 (±0.0019)
liver	0.15	0.18	9.6 ± 3.2%	0.30 ± 0.08	0.15–2.4	0.980	*y* = 0.7524 (±0.0381)*x* + 0.0238 (±0.0014)
kidney	0.15	0.17	7.5 ± 2.3%	0.30 ± 0.06	0.15–2.4	0.984	*y* = 0.7124 (±0.0499)*x* + 0.0103 (±0.0007)
fat	0.10	0.12	9.1 ± 3.6%	0.30 ± 0.06	0.10–2.4	0.993	*y* = 0.8023 (±0.0321)*x* + 0.0224 (±0.0009)
eggs	0.10	0.14	11.2 ± 3.2%	0.30 ± 0.06	0.10–2.4	0.987	*y* = 0.7977 (±0.0399)*x* + 0.0011 (±0.0001)
honey	0.10	0.13	10.3 ± 3.2%	0.30 ± 0.08	0.10–2.4	0.981	*y* = 0.8124 (±0.0366)*x* + 0.0130 (±0.0007)
sausage	0.10	0.12	8.3 ± 4.4%	0.30 ± 0.07	0.10–2.4	0.997	*y* = 0.8634 (±0.0518)*x* + 0.0330 (±0.0019)
ham	0.10	0.12	6.4 ± 2.6%	0.30 ± 0.05	0.10–2.4	0.992	*y* = 0.8536 (±0.0427)*x* + 0.0322 (±0.0019)
headcheese	0.10	0.12	9.2 ± 4.3%	0.30 ± 0.06	0.10–2.4	0.988	*y* = 0.8750 (±0.0525)*x* + 0.0354 (±0.0021)
intestines	0.10	0.12	9.3 ± 5.0%	0.30 ± 0.06	0.10–2.4	0.997	*y* = 0.8623 (±0.0321)*x* + 0.0302 (±0.0012)
aquaculture products	0.10	0.13	6.3 ± 3.6%	0.30 ± 0.06	0.10–2.4	0.996	*y* = 0.8670 (±0.0324)*x* + 0.0352 (±0.0011)
royal jelly	0.10	0.13	9.9 ± 4.3%	0.30 ± 0.10	0.10–2.4	0.984	*y* = 0.8029 (±0.0962)*x* + 0.0123 (±0.0012)
mead	0.10	0.12	7.3 ± 3.4%	0.30 ± 0.06	0.10–2.4	0.987	*y* = 0.8122 (±0.0461)*x* + 0.0098 (±0.0009)

**Table 2:** Parameters obtained for the calibration curve.

Matrix	Repeatability (RSD_r_, %)	Within-Lab Reproducibility (RSD_wR_, %)	Apparent Recovery (%)	Repeatability (RSD_r_, %)	Within-Lab Reproducibility (RSD_wR_, %)	Apparent Recovery (%)	Repeatability (RSD_r_, %)	Within-Lab Reproducibility (RSD_wR_, %)	Apparent Recovery (%)
		0.30 µg kg^−1^			0.45 µg kg^−1^			0.60 µg kg^−1^	
butter	6.5 ± 4.0	6.6 ± 4.2	103.2 ± 2.8	6.2 ± 3.8	6.6 ± 4.0	102.3 ± 3.1	6.5 ± 3.2	6.3 ± 3.9	103.4 ± 2.3
sour cream	4.7 ± 3.6	5.1 ± 4.1	102.2 ± 1.5	4.6 ± 3.9	5.0 ± 4.0	101.9 ± 3.5	4.3 ± 3.4	4.8 ± 3.8	101.6 ± 2.9
curd cheese	6.2 ± 2.3	6.5 ± 2.1	98.2 ± 3.4	6.0 ± 2.1	6.3 ± 2.3	98.7 ± 3.1	6.0 ± 2.0	6.0 ± 1.9	98.7 ± 2.6
whey	3.8 ± 5.1	5.6 ± 4.3	101.3 ± 2.4	3.7 ± 4.5	5.2 ± 4.1	102.3 ± 3.1	3.6 ± 4.9	4.3 ± 3.3	102.9 ± 3.1
milk	8.0 ± 2.1	8.3 ± 2.6	98.8 ± 2.5	7.5 ± 2.4	7.8 ± 2.9	98.2 ± 2.9	7.7 ± 2.3	7.3 ± 3.2	99.3 ± 3.2
water	4.7 ± 3.4	5.4 ± 4.1	103.5 ± 1.8	3.5 ± 3.3	4.5 ± 3.9	102.7 ± 2.8	4.4 ± 3.4	4.4 ± 4.0	102.9 ± 2.6
feed	9.1 ± 4.4	13.1 ± 5.3	91.1 ± 2.8	8.9 ± 4.2	9.1 ± 5.6	94.1 ± 3.2	8.6 ± 4.0	10.1 ± 4.8	94.2 ± 4.6
urine	8.2 ± 3.9	8.9 ± 4.3	108.0 ± 4.3	7.3 ± 3.9	8.3 ± 4.4	103.5 ± 3.3	8.0 ± 3.2	8.7 ± 3.9	106.1 ± 3.2
plasma	8.3 ± 3.5	8.6 ± 4.1	95.0 ± 6.8	7.4 ± 3.6	8.4 ± 3.2	97.2 ± 5.3	7.6 ± 3.4	8.0 ± 3.3	96.3 ± 4.3
muscle	6.4 ± 3.2	8.4 ± 3.2	105.1 ± 4.1	6.2 ± 3.1	8.1 ± 3.4	104.3 ± 3.8	6.0 ± 3.5	7.9 ± 3.4	103.2 ± 3.8
liver	9.5 ± 4.6	10.1 ± 6.3	93.1 ± 5.4	8.3 ± 4.1	9.3 ± 4.4	94.5 ± 5.0	8.7 ± 4.0	9.5 ± 5.4	94.2 ± 3.4
kidney	7.3 ± 3.5	8.5 ± 3.1	95.2 ± 4.2	7.1 ± 3.2	8.0 ± 3.2	96.1 ± 3.2	6.3 ± 3.5	8.0 ± 3.3	96.3 ± 3.6
fat	5.5 ± 4.9	6.7 ± 4.3	104.3 ± 2.4	5.0 ± 4.3	6.2 ± 4.1	104.8 ± 2.7	5.2 ± 4.6	6.3 ± 3.3	102.9 ± 3.1
eggs	7.0 ± 5.3	9.2 ± 5.6	94.0 ± 2.3	6.3 ± 4.2	8.8 ± 5.2	96.2 ± 2.3	6.2 ± 4.3	8.3 ± 5.4	96.2 ± 3.3
honey	9.2 ± 4.3	10.7 ± 3.7	96.7 ± 3.3	7.8 ± 4.1	9.9 ± 3.3	95.7 ± 3.3	8.7 ± 4.5	10.1 ± 3.8	97.6 ± 2.8
sausage	9.3 ± 4.3	10.6 ± 3.3	93.1 ± 3.7	8.3 ± 3.6	10.0 ± 3.2	95.3 ± 2.7	9.0 ± 4.7	10.0 ± 3.6	95.3 ± 3.2
ham	5.3 ± 3.4	6.5 ± 3.4	94.1 ± 2.8	5.3 ± 3.0	6.1 ± 3.0	95.2 ± 3.3	5.5 ± 3.1	6.0 ± 3.1	95.2 ± 3.3
headcheese	5.6 ± 3.2	6.9 ± 3.6	95.3 ± 4.3	5.2 ± 3.2	6.4 ± 3.1	96.4 ± 3.3	5.1 ± 3.5	6.7 ± 3.6	96.4 ± 4.0
intestines	6.5 ± 4.0	6.5 ± 4.0	93.3 ± 5.0	5.7 ± 3.7	6.1 ± 4.0	94.4 ± 4.6	5.8 ± 3.3	6.2 ± 3.2	96.0 ± 4.3
aquaculture products	5.2 ± 3.4	6.3 ± 4.4	105.0 ± 2.9	4.1 ± 3.3	5.9 ± 4.1	104.3 ± 3.9	4.0 ± 3.6	6.3 ± 4.0	103.8 ± 3.9
royal jelly	7.9 ± 4.2	11.1 ± 5.1	93.1 ± 5.2	7.9 ± 4.3	10.3 ± 3.9	95.5 ± 4.2	7.0 ± 4.2	9.2 ± 4.1	96.4 ± 4.0
mead	4.2 ± 2.3	5.7 ± 4.3	97.4 ± 3.8	4.0 ± 2.5	5.2 ± 3.9	96.4 ± 2.8	4.6 ± 2.7	5.4 ± 3.3	98.0 ± 2.8
